# Probability of Detecting Marine Predator-Prey and Species Interactions Using Novel Hybrid Acoustic Transmitter-Receiver Tags

**DOI:** 10.1371/journal.pone.0098117

**Published:** 2014-06-03

**Authors:** Laurie L. Baker, Ian D. Jonsen, Joanna E. Mills Flemming, Damian C. Lidgard, William D. Bowen, Sara J. Iverson, Dale M. Webber

**Affiliations:** 1 Department of Biology, Dalhousie University, Halifax, Nova Scotia, Canada; 2 Department of Mathematics and Statistics, Dalhousie University, Halifax, Nova Scotia, Canada; 3 Population Ecology Division, Bedford Institute of Oceanography, Dartmouth, Nova Scotia, Canada; 4 VEMCO Ltd., Halifax, Nova Scotia, Canada; University of Aveiro, Portugal

## Abstract

Understanding the nature of inter-specific and conspecific interactions in the ocean is challenging because direct observation is usually impossible. The development of dual transmitter/receivers, Vemco Mobile Transceivers (VMT), and satellite-linked (e.g. GPS) tags provides a unique opportunity to better understand between and within species interactions in space and time. Quantifying the uncertainty associated with detecting a tagged animal, particularly under varying field conditions, is vital for making accurate biological inferences when using VMTs. We evaluated the detection efficiency of VMTs deployed on grey seals, *Halichoerus grypus*, off Sable Island (NS, Canada) in relation to environmental characteristics and seal behaviour using generalized linear models (GLM) to explore both post-processed detection data and summarized raw VMT data. When considering only post-processed detection data, only about half of expected detections were recorded at best even when two VMT-tagged seals were estimated to be within 50–200 m of one another. At a separation of 400 m, only about 15% of expected detections were recorded. In contrast, when incomplete transmissions from the summarized raw data were also considered, the ratio of complete transmission to complete and incomplete transmissions was about 70% for distances ranging from 50–1000 m, with a minimum of around 40% at 600 m and a maximum of about 85% at 50 m. Distance between seals, wind stress, and depth were the most important predictors of detection efficiency. Access to the raw VMT data allowed us to focus on the physical and environmental factors that limit a transceiver’s ability to resolve a transmitter’s identity.

## Introduction

Electronic tracking and telemetry data have greatly improved our knowledge about the ecology of many marine species at the individual and population levels [1]. However, few studies have used these methods to investigate the nature of interactions between individual animals. Interactions among conspecifics and between species shape both social and ecosystem structures, and can affect population growth rates, distribution, diversity, and gene flow [2], [3]. Studies of predator-prey, competitive and social interactions in marine species have largely been inferred from experiments [4], diet sampling [5], multi-species time series analyses [6], , or direct observation [8]. These studies are often limited to accessible habitats (e.g. the intertidal, haul out sites) and may not provide insight at the individual level (e.g. time series analysis). Acoustic telemetry can overcome some of these shortcomings by providing information about interactions at the level of individuals from inaccessible marine environments, see Barnet et al. [9] and Barnet and Semmens [10] who simultaneously tracked predator and prey.

The deployment of dual transmitting and receiving acoustic Vemco Mobile Transceivers (VMT, www.vemco.com) and satellite-linked GPS tags or geolocation tags [11] on large marine vertebrates provides an opportunity to understand species interactions in space and time. The VMT is a hybrid acoustic tag, housing a 69 kHz coded transmitter and a 69 kHz monitoring receiver (similar to the VR2W). Whereas arrays of stationary acoustic receivers are often necessarily confined to continental shelf areas (e.g. [12]), the deployment of VMTs on marine animals provides the ability to extend detection ranges of conspecific and other marine species to biologically interesting regions that may be missed by fixed arrays. The dual transmitter and receiver capabilities of the VMT create a mobile receiving station by which non-surfacing acoustic-tagged organisms, such as fish, can be detected. With these data we have the capacity to better understand the role of predators in ecosystems and to improve our understanding of their interactions with commercial fish stocks and fish species of conservation concern.

To interpret interactions between two organisms we must accurately describe the interaction locations, duration, and frequency. At the most basic level, this relies on knowing whether or not a tagged organism is present. Quantifying the probability of detecting a tag if it is near a given receiver, particularly under changing field conditions, is vital for making accurate biological inferences when using these VMTs (e.g. Argos, [13]; geolocation, [14]). In general, the probability of detecting a transmitter depends on the distance the transmitter is from the receiver, the properties of the medium and transmission (e.g. sound frequency), and the presence of physical obstructions and noise [15]. Sound intensity attenuates with the square of the range according to geometric spreading of the sound in water [15]. Therefore the distance a transmission travels in the ocean depends strongly on the sound frequency of the signal and characteristics of the propagation medium (i.e. sea water composition). Detection probability can also be affected if parts of the transmission are masked by background noise or distorted (e.g. changes in transmission frequency).

Changes in detection efficiency may occur in response to changes in oceanographic and environmental conditions: wind stress [16], [17]; water column stratification [18], [19]; water density [20], [18]; bottom topography [21]. Detection efficiencies have been quantified using a range of approaches: boat based, diver based, fixed sentinel tags, fixed tag with receiver at set distances, post-analysis, single tag at different distance, etc. [22]. While these studies provide valuable data on detection ranges, they cannot fully describe conditions experienced off-shore, and therefore cannot be expected to assess the performance of the VMT when deployed on a free-ranging marine animal. Our case study is distinct from standard acoustic studies, where only the tag is in motion; in our case both the tag and receiver are in motion. The importance of understanding how a tagged marine animal’s behaviour affects tag performance is therefore increased. Differences between VMTs may arise because some individuals spend a greater proportion of their time in noisier locations or near complex geomorphology, which may lead to more obstructed transmissions [23] than in other locations. Understanding these behavioural patterns and how they differ seasonally, by age, sex, and physiological state is of the utmost importance.

Pinnipeds are well suited for testing the performance of VMTs. Their frequent return to the surface provides highly accurate GPS locations. Grey seals (*Halichoerus grypus*) fitted with VMTs are known to interact frequently with each other [24], and exhibit high site fidelity, making them easy to recapture to retrieve archived data. Evaluating VMTs when deployed on grey seals provides an opportunity to assess the efficiency of VMTs under realistic behavioural and environmental conditions. Here, we define detection efficiency as how well VMTs are able to detect another VMT transmitter (i.e. with what probability) within a defined range.

We conducted two analyses of detection efficiency of VMTs deployed on grey seals using post-processed detection data (complete transmissions) and summarized raw VMT data (complete and incomplete transmissions), to explore the effect of environmental factors: wind stress, distance between VMTs, and temperature and depth gradients. The raw VMT data consists of a record of all acoustic pings (the smallest sound unit) recorded by the VMT receiver, and differs from the post-processed detection data in that it contains records of incomplete transmissions in addition to complete transmissions (confirmed detections) as well as pings from environmental and anthropogenic sources. Vemco provided us with summarized raw data for four VMTs consisting of acoustic pings classified by the time intervals between them and summed for each 10-minute period.

We evaluated the detection efficiency of VMTs, using calculated distances (based on GPS locations) between seals to generate a series of instances when detections are likely to have occurred. Access to the summarized raw VMT data allowed us to focus on the physical and environmental factors that limit a receiverability to resolve a transmitteridentity.

## Materials and Methods

### Ethics Statement

This research was conducted in accordance with guidelines for the use of animals in research [25] and of the Canadian Council on Animal Care. The research protocol for deployment of tags on grey seals was approved by the University Committee on Laboratory Animals, Dalhousie University’s animal ethics committee (animal care protocol: 08–088) and the Department of Fisheries and Oceans, Canada (animal care permit: 10–65).

### Study Site

**Figure 1:**
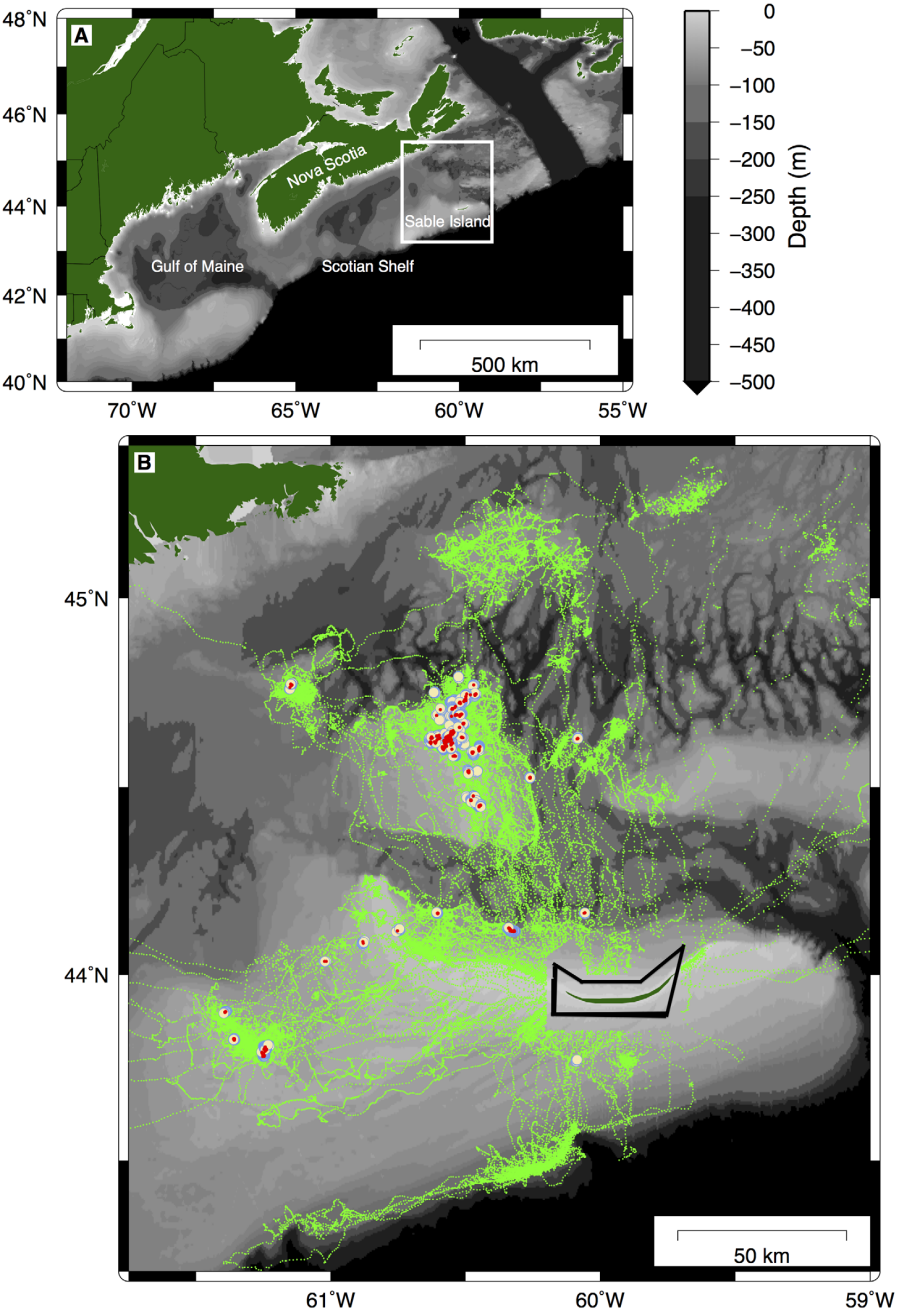
Study area and seal tracks. Nova Scotia and the Scotian Shelf (A) with the study area showing GPS tracks (green) and VMT expected (white) and observed (red) detections (B). The main shallow banks in the region are outlined with their 100 m isobaths (grey).

The study was conducted between 8 September 2010 and 17 January 2011 on Sable Island, Nova Scotia, Canada (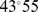, 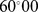 and the Eastern Scotian Shelf in the northwest Atlantic Ocean (Figure 1). Sable Island is an important breeding site for grey seals [26] and the Eastern Scotian Shelf is an important foraging area [24], [27].

### Study Animals

Seventeen adult grey seals, *Halichoerus grypus* (Fabricius, 1791), selected from a pool of known-age adults were captured between 8 and 18 September 2010 on Sable Island and fitted with a VHF transmitter (164–165 MHz, www.atstrack.com), GPS satellite-linked tag (MK10-AF, www.wildlifecomputers.com) and a VMT according to the methods described in Lidgard et al. [24]. Briefly, the VHF and GPS tags were attached just below the neck to maximize the time the GPS tag spent above water where it could record the satellites in range. The VMT was attached to the lower back of the seal to increase the time the VMT spent in the water transmitting and receiving detections and to reduce electrical interference with the satellite tag. The GPS tag was programmed to collect light intensity, depth (m), and temperature (°C) every ten seconds and to record a GPS location every 15 minutes. GPS attempts were suspended when the unit was dry more than 20 minutes or when a location had been attained.

Peak sensitivities for hearing in phocids are between about 10 and 50 kHz with a high frequency limit of 100 kHz [28]. It is likely that seals could hear the 69 kHz VMT transmissions, given the power output of the transmitters (146–149 dB re 1 *µ*Pa SPL 1 m) [29]. However, we did not observe any differences in behaviour: seals in this study exhibited similar foraging and breeding patterns to seals previously tagged with satellite transmitters without an acoustic tag [27], [30], [31]. Ambient background noise, reflection and refraction of the signal, and habituation to the signal over time, make it unlikely that seals could localize other VMT tagged seals. Individuals were recaptured on Sable Island during the subsequent breeding season (December 2010 to January 2011) and their tags retrieved (median deployment period = 112 d range = 92–121 d).

### Post-processed Detection Data vs. Summarized Raw VMT Data

**Figure 2:**
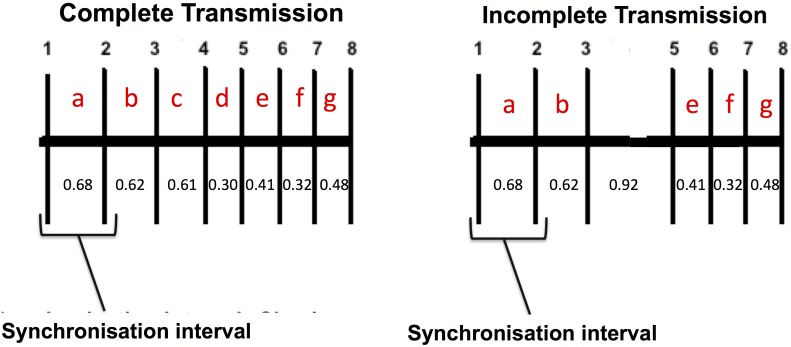
Complete vs. incomplete transmission. VMT transmissions comprise a series of 8 acoustic pings. Each oustic ping stringontains a synchronization interval (between the first two pings), used to identify acoustic-tag transmission format, followed by a series of pings unique to each individual tag. Intervals between 0.30–0.70 s correspond to consecutive pings. An interval between 0.70–1.50 s may indicate that one ping (of duration 0.01 s) is missing, e.g. time interval of 0.92 s in the incomplete transmission diagram. All 8 acoustic pings must be received for a detection to be recorded.

VMTs are coded transmitters, meaning they transmit a sequence of pings that form an acoustic code unique to each individual VMT. VMTs are programmed to transmit an acoustic code on an irregular schedule, every 60 to 180 seconds. During each code transmission the VMT turns off its receiver for approximately 3.5 s, to avoid receiving echos from its own transmission that could interfere with code validation, and records the date and time of the transmission. Each code transmission comprises a sequence of eight acoustic pings (acoustic code). Each acoustic code begins with a synchronisation interval (sync)– the time between the first two acoustic pings– that identifies the transmission format. The series of acoustic pings that follows creates the unique identification code; the interval between each of the eight acoustic pings creates the unique identification code (Figure 2). A checksum is applied to the entire acoustic code to identify the legitimacy of the transmission. Hereafter, we use the terms transmission and acoustic code synonymously.

Post-processed detection data, available to all VMT users, comprises the complete received 69 kHz transmission– which may originate from a VMT or other 69 kHz transmitter– and a daily summary of the total number of acoustic pings, syncs, and rejected false detections. Received complete transmissions (detections), in VMT memory, comprise a date-time stamp and the identities of the transmitting and receiving acoustic tags. False detections are identified by VEMCO, using proprietary software, and removed from the dataset upon VMT retrieval. False detections may result from the collision of codes from other active transmitters that either generate a code that does not exist or an existing code that is known to be present elsewhere (e.g. tags deployed on freshwater species or on non-migratory species in other ocean basins).

The summarized raw VMT data is different from the post-processed detection data in that it includes all acoustic pings received by the transmitter, including those from incomplete transmissions. Acoustic pings may originate from a variety of sources such as other VMTs, acoustic transmitters and abiotic and biotic noise. Acoustics pings originating from VMTs and other VEMCO transmitters may be distinguished from background noise by the signature intervals between each ping in their acoustic codes (Table 1). VMTs are programmed such that consecutive acoustic pings in an acoustic code occur between 0.30 s and 0.70 s. Acoustic pings may also occur at intervals within 0.70 s and 1.50 s in cases where one or more acoustic pings in a code are missing (Figure 2). We therefore defined the range at which probable VMT pings occur as 0.30 s to 1.50 s. Acoustic pings occurring at intervals between 0.26 s and 0.30 s are thought to indicate possible echos, multipath transmissions, or transmission collisions. Acoustic pings occurring at intervals greater than 1.50 s are likely the result of environmental noise or are cases where VMTs are near their acoustic range limit.

**Table 1:** Criteria used to determine ping origins.

Interval Length	Description
0.26–0.29 s	Possible echos or multipath transmissions
**0.30–0.70 s**	Interval range between **consecutive pings**
**0.71–1.50 s**	Interval range between **1 or more skipped pings**
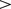 1.50 s	Spurious pings or 3 or more skipped pings

*Ping origins deduced from intervals between consecutive pings.

### Track Data and Expected vs. Observed Detections

We determined GPS locations by analyzing archival GPS data from each tag using software from the manufacturer. To be considered accurate, locations had to be acquired from 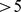 satellites with a residual error 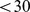 m [32], [33].

To link encounters between instrumented seals to locations interpolated at 3 min intervals from the seal tracks, clocks in the VMT and GPS tags were synchronized upon deployment and time corrected upon retrieval based on the respective clock drift calculated from GPS and VMT tags over the deployment time [24]. Distances between seals (m) were calculated from the 3-min interpolated locations.

**Figure 3:**
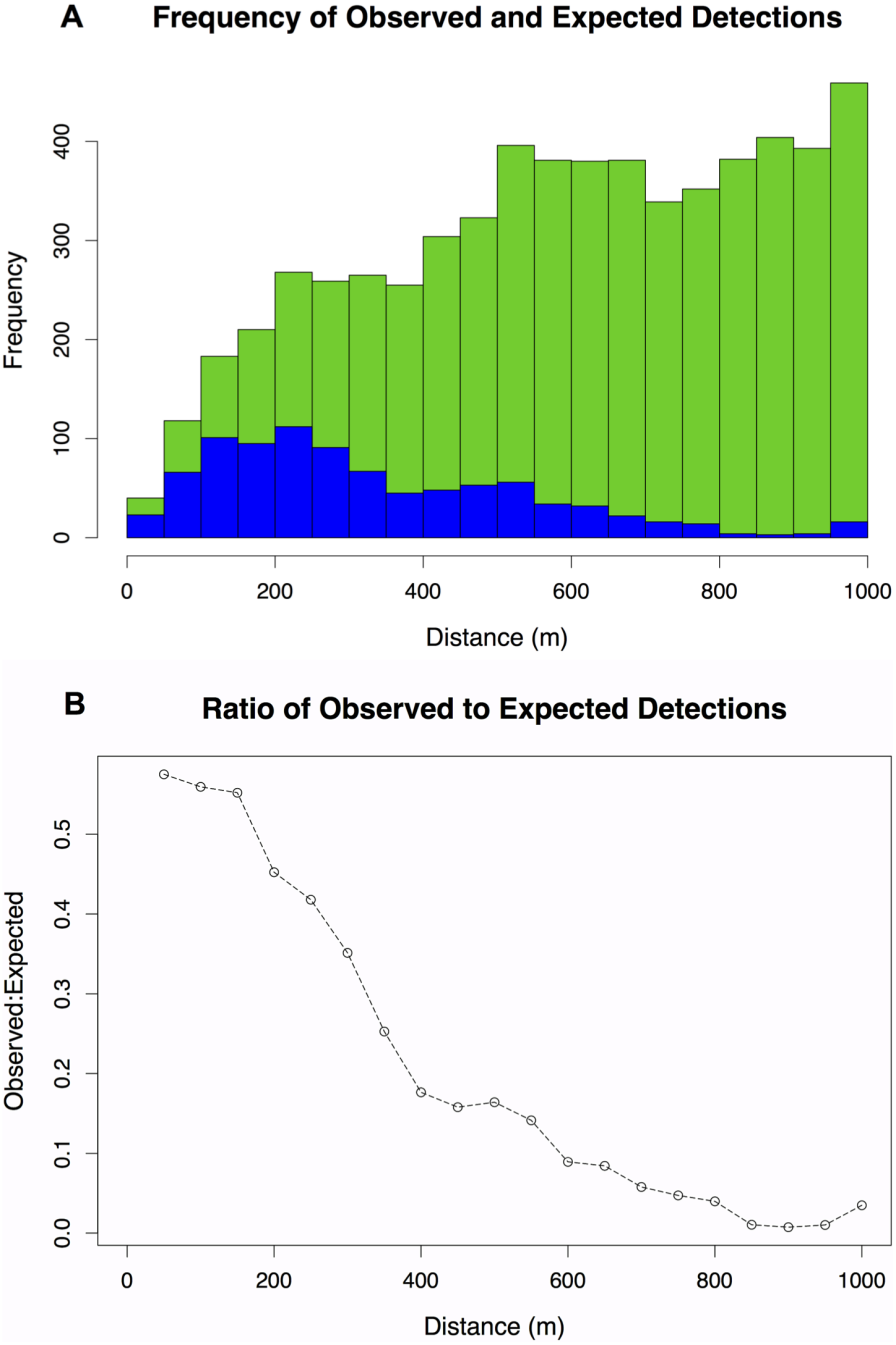
Density and ratio of detections. A. Density of observed (blue) and expected detections (green) with distance. B. Plot of the ratio of observed to expected detections.

Each seal’s travel rate (m/s) was calculated using the original archival GPS location data. We matched these estimates to the respective transmitting and receiving VMTs using a date-time stamp. We assumed expected detections to occur every 180 s, based on tag specifications (every 60–180 s), when two VMTs encountered each other. We operationally defined an expected encounter as occurring when the VMTs were within 100–700 m of one another. We used 100 m as the lower limit of this range to avoid a decreased probability of detection, which may sometimes occur at close encounter ranges. We used 700 m as the upper limit of our range based on the manufacturers specifications and inspection of our detection data (Figure 3).

Despite being within range of VMTs that recorded data, two VMTs (66487, 66548) failed to record any detections, and one VMT (66494) was only recorded once by another VMT. Closer inspection of the seal tracks associated with these VMTs indicated they were spatially peripheral to the majority of the VMT-tagged seals, but still within range of certain known working VMTs. We excluded these non-functioning VMTs (66487, 66548, 66494). There were also confounding elements that could have affected the summarized raw VMT and post-processed detection data around the VMT deployment point, Sable Island. VMTs do not record signals out of the water; therefore it is important to exclude any periods the seal is out of the water from the analysis. Close to the island, it was difficult to determine if a VMT-tagged seal was out of the water if these durations were shorter than the wet-dry sensors on the GPS tag could detect. Furthermore, due to the shallow bathymetry and thus high noise disturbance around the island, we expected the capability of the VMT to record transmissions to be compromised. Thus, detection data around Sable Island were removed prior to analyses (see polygon outlined in Figure 1B).

### Conversion Efficiency

Vemco provided summarized raw VMT data for four of the VMTs (66556, 66504, 66555, 66541). From these data we calculated the VMT conversion efficiency. Conversion efficiency was defined as the ratio of acoustic pings translated into detections (complete VMT transmissions) to those received (complete and incomplete VMT transmissions, Figure 2).

### Statistical Model and Environmental Variables

We used a generalized linear model (GLM) with a negative binomial distribution to model VMT detection and conversion efficiency, where the response variable was the number of observed detections from new encounters in a 12 h period. New encounters were identified as detections (expected or observed) occurring when there was at least a 30 min interval between consecutive detections for a defined pair of seals. The number of expected detections in each 12 h period was included in the model as an offset term to account for the time VMT-tagged seals spent near each other.

Conversion efficiency was evaluated by modelling the number of acoustic pings from complete VMT transmissions (observed detections×8 pings), including the total number of pings from VMTs received (pings occurring at intervals between 0.3–1.5 s) in 10 min intervals as an offset.

### Environmental Variables

Environmental variables were selected according to their relevance to sound propagation on the Scotian Shelf and their availability (Table 2). To avoid temporal and spatial scale mismatches, most variables were limited to those that we could collect from the MK 10-AF tags which sampled every 10 seconds and at the seal’s exact location. Temperature (°C) and depth gradients (m) between the transmitting and receiving seals were included in the model to test for the effect of water stratification and density changes. The directional (positive or negative) difference in depth and temperature was included because the direction of signal travel with respect to the temperature or depth gradient affects sound transmission differently. Horizontal distance (m) was included in the model to represent detection range.

**Table 2:** Environmental variables explored in VMT efficiency analyses.

Variable	Description
negtempdif	Directional temperature difference ( 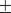  C)
mindepth	Depth of the shallowest seal (m)
distance	Horizontal distance between seals (km)
negdepdif	Directional depth difference ( 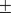 m)
travel rate	Travel rate of the receiving seal (m/s)

*Description of environmental variables tested in VMT efficiency analyses.

Wind stress (N/m^2^) was included in the model to test the effect of increased noise and changes in the air-sea interface through the introduction of air bubbles. Wind stress (N/m^2^) was calculated from hourly estimates of wind speed on Sable Island (Department of Fisheries and Oceans, Canada) in MATLAB (MathWorks, Inc.), using the function stresslp.m (package: air and sea) following Large and Pond [34]. We hypothesized that the effect of noise and/or air bubbles generated by wind stress would be greatest at the surface; we therefore tested for a possible interaction between wind stress (N/m^2^) and the depth of the shallowest seal (m) in the model. Seal identity was included as a factor to account for variation in VMT performance and differences in seal behaviour and movement patterns. Travel rate (m/s) was included to describe the seal’s horizontal movement rates.

### Model Selection

Terms in the model were added and subtracted using forward and backward selection [35]. Variable selection was based on hypothesis testing (p-values) and by comparing the pseudo adjusted R^2^ calculated from the residual and null deviance of the model. Residual diagnostics were examined to determine goodness of fit. To explore how sensitive the results were to the subsample distance range, we explored the data subset by distance ranging from 100–250 m, 100–400 m, and 100–700 m. This was done to control for varying amounts of time spent by seals at different distances from one another.

## Results

**Figure 4:**
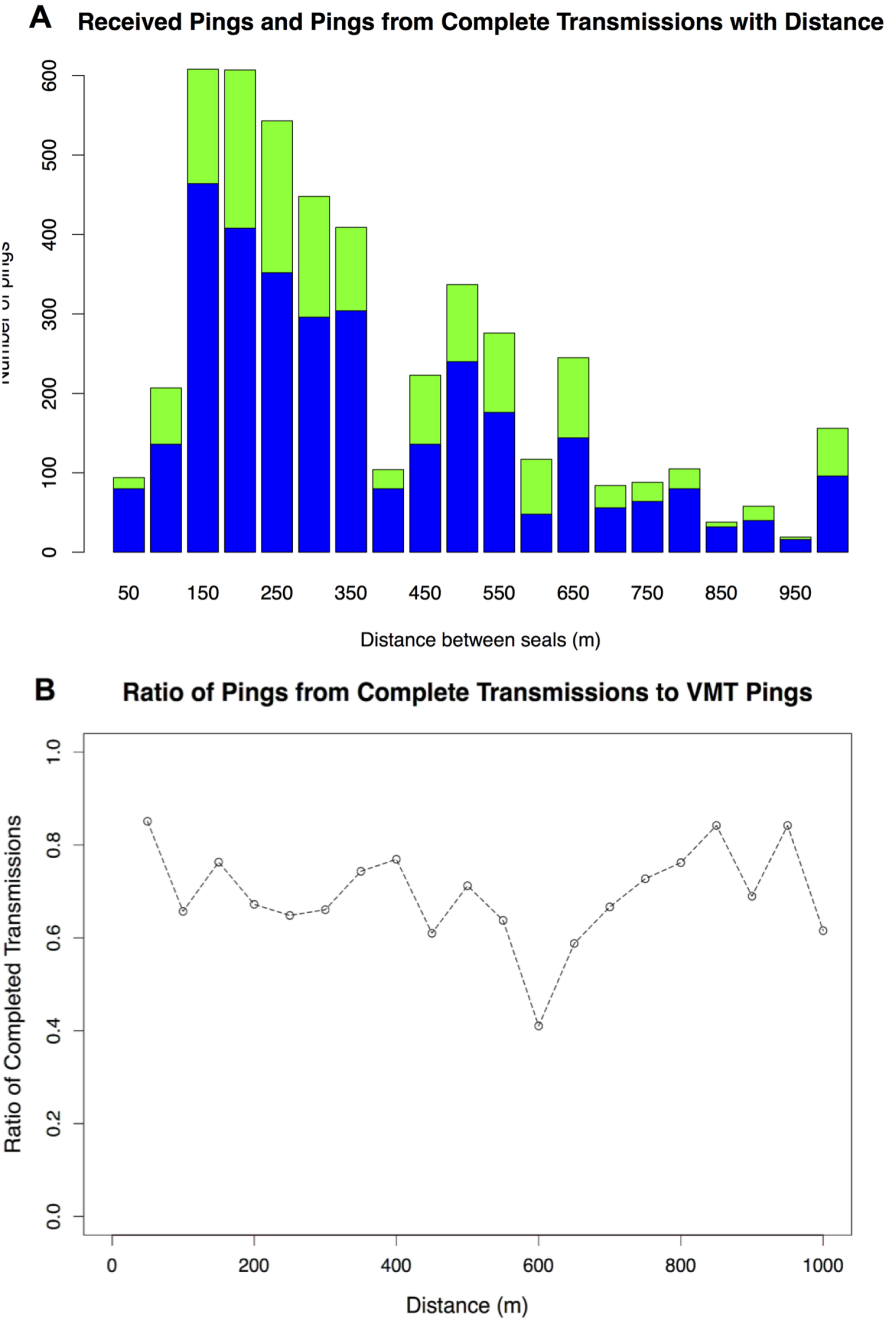
Density and ratio of VMT acoustic pings. A. Density of VMT acoustic pings received (green) and acoustic pings from VMT transmissions (blue) with distance. B. Plot of the ratio of pings from complete transmission to VMT pings received.

All 17 deployed VMT and GPS tags were recovered from seals upon their return to Sable Island during the breeding season. GPS locations were acquired with a median of 9 satellites (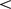
15 m residual error). A total of 1,168 detections were recorded, occurring at distances between 4 m and 1880 m (median = 320 m, mode = 250 m). Fewer detections occurred at both close range and and beyond 500 m. 60% of all detections occurred when the VMTs were within 500 m of one another (Figure 3A). We observed a decrease in the proportion of observed vs. expected detections with increased distance (Figure 3B). Only about half of the expected detections were recorded even when two VMT-tagged seals were estimated to be within 50–200 m. At a separation of 400 m, only about 15% of expected detections were recorded. The summarized raw VMT data provided a clearer picture of whether any part of a transmission was received with distance (Figure 4): the ratio of pings from complete transmission to pings from complete and incomplete transmissions fluctuated around 70%, with a minimum of around 40% at 600 m and a maximum of about 85% at 50 m (Figure 4).

### Model 1: Expected and Observed Detections

**Figure 5:**
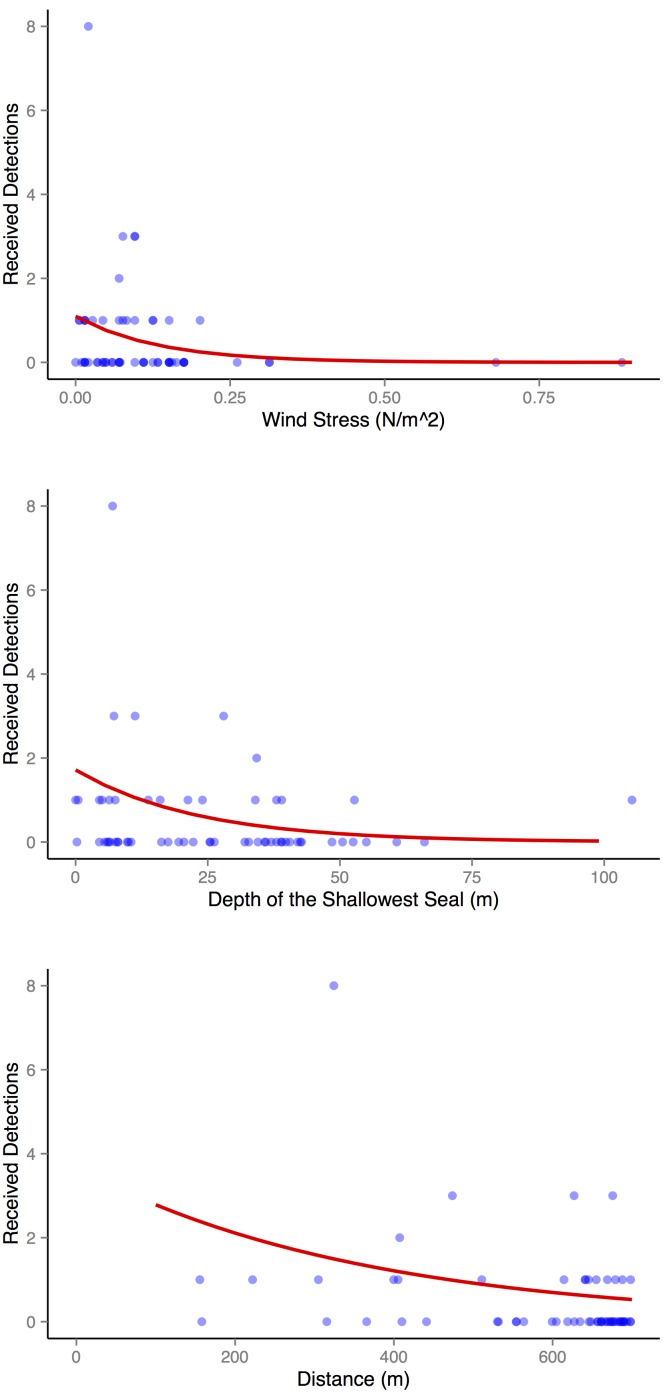
Factors affecting detection efficiency. The predicted effect on detection efficiency of the significant variables (red line): wind stress, minimum depth, and distance. Fitted values (observed detections offset by expected detections) as points. Points: dark blue indicates high intensity, light blue indicates low intensity.

The best model explained 35.7% of the variability in the detection efficiency. The probability of detection decreased with increasing distance between seals (−2.77, SE: 0.64), wind stress (−7.40, SE: 1.87), and depth of the shallowest seal (−0.03, SE: 0.01), (Figure 5).

### Model 2: Conversion Efficiency

**Figure 6:**
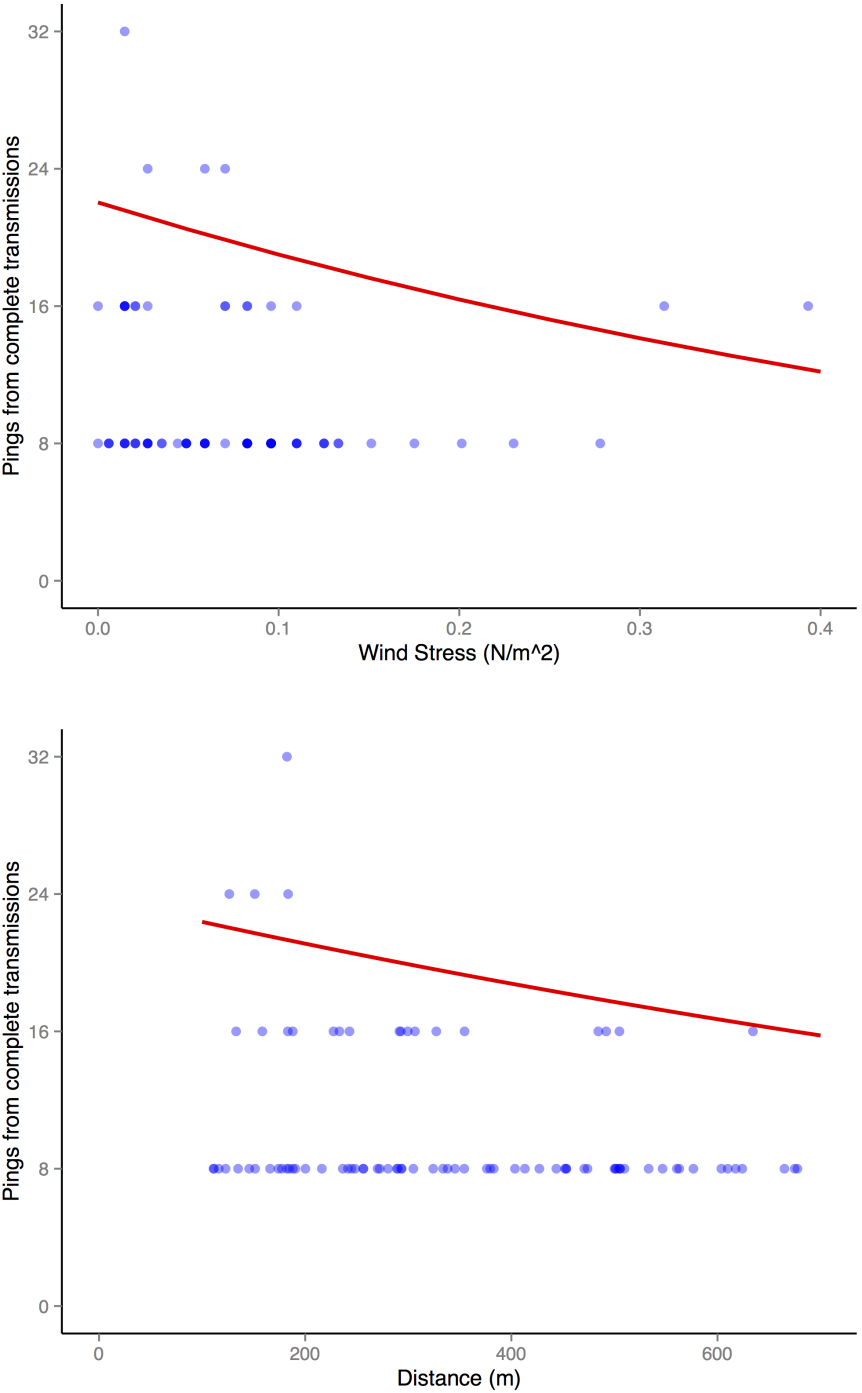
Factors affecting conversion efficiency. The predicted effect on conversion efficiency of the significant variables (red line): wind stress and distance. Fitted values (VMT acoustic pings from complete transmissions offset by total VMT acoustic pings received) as points. Points: dark blue indicates high intensity, light blue indicates low intensity.

Wind stress (−1.59, SE: 0.35) and distance (−0.54, SE: 0.14) were both important predictors of conversion efficiency. Conversion efficiency decreased with increasing wind stress and increasing distance (Figure 6). Wind stress had the most significant effect on detection efficiency.

### Sensitivity of Detection Efficiency to Distance Range

The results from each data subset were generally consistent with those of the main analyses. When encounters were defined at the 100–400 m range, results were consistent with the main analysis (100–700 m), but when encounters were defined at the 100–250 m range depth of the shallowest seal did not have a significant effect on detection efficiency. The signs and coefficients of model terms were conserved across distance ranges. The pseudo adjusted R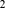 values were 19.5%, 28.1%, and 35.72% for the interval ranges: 100–250 m, 100–400 m, and 100–700 m respectively. These changes in explanatory power are likely the result of the increased influence of distance on decreases in detection efficiency.

## Discussion

While it is relatively easy to ascertain if a tagged animal is present (true positive), it is more difficult to determine with certainty that it is absent (true negative) as it could be present but not detected (false negative). Quantifying the proportion of VMT transmissions that are not received and determining to what extent this is due to physical and environmental factors and the behaviour of the tagged animals, is vital to form accurate ecological conclusions from VMT data. Without an appreciation of these issues, these effects may lead to erroneous inferences.

We present one of the first studies to investigate the detection efficiency of acoustic VMT receivers deployed on marine animals and to analyze detection efficiency using summarized raw VMT data. Wind stress, depth of the shallowest seal, and distance between seals were significantly correlated with VMT performance. The summarized raw VMT data allowed us to determine the extent to which within-range VMTs are successfully detected and provided a clearer picture of whether any part of a VMT transmission is received. The ratio of VMT pings from complete transmissions to VMT pings received fluctuated around 70% with a minimum of around 40% at 600 m and a maximum of about 85% at 50 m. This shows a vast improvement when compared with at best 50% of expected detections received between 50–200 m, dropping to 15% at 400 m when using only the post-processed detection data. Examining conversion efficiency (the ratio of complete transmissions to all transmissions received) provides additional insight into VMT detection efficiency by focusing on factors that limit a transceiver’s ability to resolve a transmitter’s identity.

To date, GPS tags provide the best location estimates for *in situ* studies of this nature. GPS locations were obtained with a small residual error (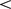
15 m) [32], resulting in little uncertainty in the GPS locations and subsequently, little uncertainty in the actual detection distances observed. Therefore, although it is possible for the seals to be 60 m closer or further away than that reported, the chance of this occurring are low.

### Environmental Factors Affecting VMT Performance

Distance between seals was a significant predictor of detection and conversion efficiency. In both cases, the probability of detection or conversion decreased with distance as expected. Detection range has long been identified as an important factor affecting the detection of acoustic tags [20]. Detection probability is hypothesized to decline proportionally to the decline in sound intensity, which is a combination of geometric and exponential decline due to sound spreading and attenuation resulting from water viscosity [15]. However, the exact shape of this relationship is unknown and modelling approaches vary. We were unable to resolve the shape of this relationship from our data due to its observational nature. However, results from our sensitivity analysis illustrate that the detection range, assumed *a priori*, did not affect the relationships observed.

We also observed a decrease in detection efficiency and conversion efficiency with increasing wind stress. Wind stress can introduce noise as well as air bubbles into the marine environment. Noise makes it difficult to distinguish the acoustic signal above the background noise and may result in failure to detect one or more of the pings. Air bubbles absorb a sound transmission because the acoustic signal has to pass between water and air. The absence of a significant interaction between wind stress and the depth of the shallowest seal suggests that the effect of wind stress on detection efficiency is not confined to surface waters. The observed decrease in detection efficiency with increasing depth may be indicative of sound attenuation occurring as a result of bathymetric effects [21].

Despite well established effects on sound transmission, we observed no effect of the propagation medium (temperature/depth gradients) on detection efficiency [15]. Sound propagation may be absorbed and deflected when traveling through density gradients (i.e., pycnocline). The coastal currents that transport source waters to the Scotian shelf exhibit strong seasonal cycles as well as significant interannual variability [36]. The Nova Scotia current reaches a peak velocity in winter, transporting low salinity and low temperature water originating in the Gulf of St. Lawrence [37] into the inshore waters. These forces generally result in a low salinity and low temperature signature inshore that is more pronounced during winter months [36]. Temperature and depth gradients are therefore more likely to affect detection efficiency after our deployment period (September–December) from January–March, than during our deployment period.

As animal-borne acoustic telemetry evolves beyond stationary receivers, it is unclear how factors such as the orientation of the VMT with respect to the animal or the size of the animal affect VMT performance. VMTs were placed on the lower back of the seal to maximize the time the VMT spent in the water receiving and transmitting signals. The sealbody might attenuate acoustic signals being transmitted to or received from a certain direction, regardless of VMT positioning. Although this effect has not been formally investigated, it would be extremely difficult to quantify *in situ*. A tri-axial accelerometer could be deployed to measure the sealspeed and VMT orientation, however, these devices also have limitations. Controlled experiments will be needed to investigate the influence of such factors on VMT performance. Other factors known to affect detection efficiency that were not included in our model are biotic and/or anthropogenic noise, e.g. [38], [39]. These, in addition to characteristics of the seals behaviour (e.g., the animalorientation during diving), may account for some of the unexplained variation in the model.

### VMT Engineering

To interpret interactions we must accurately define their location, duration, frequency, and confidently identify legitimate periods of silence (true negative), i.e. the absence of transmissions. For a detection to occur, the VMT receiver must be able to distinguish the acoustic signal from background noise. The background noise strength is dependent on weather and the fluid environment and other sources, including anthropogenic noise [15]. Distinguishing legitimate transmissions from background noise is an important component of measuring VMT performance. Simpfendorfer et al. [23] used syncs to estimate the volume of received incomplete and complete transmissions for a given period relative to the number of recorded transmissions; however, syncs are not precise. When tag transmissions collide, syncs can be created that are not from a tag transmission; consecutive pings from different tags may create a pseudo sync interval. The use of summarized raw VMT data addresses this shortcoming by utilizing aspects of the transmission that are less susceptible to false positives. With access to the summarized raw VMT data, users can examine the interval between consecutive pings to determine their origin and thus authenticity, i.e. whether the pings arose from echoes, multi-path collisions, environmental noise or are legitimate pings from a VMT.

Observational data in the ocean are often limited due to the technological, environmental, and physical challenges that accompany data collection. These constraints make it important to maximize what can be gleaned from such data. Currently, access to the summarized raw data is not routinely available. Wider access to data of this sort will provide users with an additional indicator of their tag’s performance, and inform their analyses through the ability to identify false-negatives. In cases where the identity of the tagged individual is not pertinent, it may be sufficient to simply know that a seal was detected when part of a VMT transmission reached the VMT, even if we cannot account for the factors affecting the VMT transmission.

Without understanding the factors affecting detection efficiency, biological inferences regarding the prevalence and nature of species interactions via VMT/acoustic data will very likely be biased. For example, seasonal changes in environmental factors, that could reduce received transmissions, may be falsely attributed to seasonal changes in interaction rate. It is therefore vital that we account for changes in detection efficiency, as without this information, it is impossible to interpret what any given detection event represents.
